# Late-Diagnosed Cystic Fibrosis with Co-Occurring *CFTR* 3849+10kbC>T and 5T Variants: A Case Report

**DOI:** 10.3390/jcm15197584

**Published:** 2026-09-30

**Authors:** Paula Popovici, Adriana Mihai, Lăcrămioara Ionela Butnariu, Razvan Mihai Popovici, Alina Mariela Murgu, Elena Tarcă, Ileana Katerina Ioniuc, Tania Elena Rusu, Dana-Teodora Anton-Păduraru, Oana-Raluca Temneanu

**Affiliations:** 1 Grigore T. Popa University of Medicine and Pharmacy, 700115 Iaşi, Romania; paula.popovici@umfiasi.ro (P.P.); razvan.popovici@umfiasi.ro (R.M.P.); alina.murgu@umfiasi.ro (A.M.M.); elena.tuluc@umfiasi.ro (E.T.); ileana.ioniuc@umfiasi.ro (I.K.I.); taniaelenarusu@gmail.com (T.E.R.); dana.anton@umfiasi.ro (D.-T.A.-P.); temneanu.oana@umfiasi.ro (O.-R.T.); 2 Department of Pediatrics, Saint Mary’s Emergency Children Hospital, 700309 Iași, Romania; 3 Regional Center for Medical Genetics Iasi, Saint Mary’s Emergency Children Hospital, 700309 Iași, Romania; 4 Department of Obstetrics and Gynecology, Cuza-Voda Clinical Hospital of Obstetrics and Gynecology, 700038 Iaşi, Romania; 5 Department of Surgery II—Pediatric Surgery, Saint Mary’s Emergency Children Hospital, 700309 Iași, Romania; 6 Regina Maria Healthcare Campus, 700023 Iaşi, Romania

**Keywords:** cystic fibrosis, *CFTR*, 3849+10kbC>T, poly-T tract, residual function, CFTR modulators, elexacaftor/tezacaftor/ivacaftor, newborn screening, case report

## Abstract

**Background:** The deep-intronic variant 3849+10kbC>T preserves residual CFTR function and is recurrently associated with pancreatic sufficiency, normal or borderline sweat chloride and diagnosis beyond early childhood. A co-occurring poly-T 5T allele adds a second layer of uncertainty: its penetrance depends on the TG repeat carried in *cis*, and its contribution to the phenotype can be weighed only once its phase relative to the second variant has been established. **Case Presentation:** A boy with recurrent respiratory infections from the age of three was diagnosed with cystic fibrosis at 12 years, in 2018—four years before newborn screening became national policy in Romania. Two sweat conductivity measurements, 125 and 80 mmol/L NaCl-equivalent, together with targeted *CFTR* analysis identifying 3849+10kbC>T and a 5T allele, established the diagnosis. Phase was not determined—parental samples were not obtained and no allele-specific or long-range analysis was performed—so the two findings are reported as co-occurring rather than as demonstrably in *trans*. His elder brother, who had recurrent spontaneous pneumothorax and two negative sweat tests, was never genotyped because the test was not reimbursed. Bilateral bronchiectasis and chronic airway infection followed; inhaled colistimethate eradicated *Pseudomonas aeruginosa*, whereas *Staphylococcus aureus* persisted despite successive antibiogram-guided regimens. Access to elexacaftor/tezacaftor/ivacaftor was granted in January 2026 on the basis of the genotype. **Conclusions:** Molecular characterisation contributed to a diagnosis that rested on the combination of clinical phenotype, abnormal sweat conductivity, and *CFTR* analysis, and was, eight years later, the sole criterion for access to modulator therapy; phase and TG-repeat length remain undetermined. No follow-up data are available, therapy having been authorised but not yet started at the time of writing.

## 1. Introduction

Of the more than two thousand sequence variants recorded in *CFTR*, only a minority have been assigned a definite disease liability, and the residual-function group sits least comfortably within that classification [1]. The 3849+10kbC>T variant (c.3718-2477C>T on the MANE Select transcript NM_000492.4, also reported as c.3717+12191C>T; rs75039782; legacy intron 19 numbering, intron 22 in current nomenclature) belongs to it. Highsmith et al. described it in 1994 in patients who had pulmonary disease yet normal sweat chloride concentrations—an observation that established at the outset that its clinical expression need not follow the classical picture [2]. The variant creates a cryptic splice site and an 84-bp pseudoexon insertion; because correctly spliced transcript persists, a proportion of functional channel remains, and the resulting phenotype is typically pancreatic sufficient, with respiratory involvement that accumulates slowly.

The poly-T tract at the acceptor site of legacy intron 8 introduces a second and independent layer of variability. Groman et al. showed that the pathogenicity of 5T is not intrinsic but conditional: the length of the adjacent TG repeat determines how efficiently exon 9 is retained, TG11 behaving as a low-penetrance background and TG12 or TG13 producing substantially more exon skipping [3]. Paired with a CF-causing variant in *trans*, the same 5T allele may therefore underlie anything from an isolated CFTR-related disorder to non-classic cystic fibrosis. Absent the TG haplotype, the genotype cannot be placed on that spectrum with confidence.

Newborn screening has compressed the age at diagnosis across most of Europe from years to weeks [4,5], and the disease seen in paediatric clinics today differs accordingly from the one described in older cohorts [6]. Romania introduced screening as national policy only in July 2022 [7]. It would be convenient to attribute a late diagnosis to that gap alone, but the reasoning is weaker than it first appears. European programmes differ substantially in the trypsinogen thresholds they apply and in the variant panels they interrogate, so the sensitivity of any given protocol is a property of that protocol rather than of screening as such [8]; and infants who are pancreatic sufficient, as those carrying residual-function variants usually are, sit in the part of the distribution where trypsinogen-based algorithms perform least reliably [9]. Screening would have improved the odds. It would not have guaranteed detection.

We describe an adolescent in whom the diagnosis was reached at 12 years and modulator therapy became accessible only at 20, and we use the interval between those two events to argue that complete molecular characterisation, rather than screening alone, was the rate-limiting resource—contributing to the recognition of the disease and subsequently governing access to treatment outright.

## 2. Case Presentation

### 2.1. Presentation and Family History

The patient, a boy, had recurrent upper and lower respiratory tract infections from the age of three, treated in primary and secondary care without a unifying diagnosis being sought. The chronology of clinical events is summarised in Table 1.

His elder brother, aged 22 years at the time of the index admission, had a history of recurrent spontaneous pneumothorax and malnutrition, without other documented manifestations; the episodes preceded the index diagnosis. Two sweat tests performed in the brother before 2018 were reported to the family as negative. Neither the numerical values nor the analytical method is available to us, and the distinction between chloride titration and conductivity measurement therefore cannot be made; the result entered this family’s history as a categorical one. The reason is structural rather than accidental. The brother was an adult at the time of the index admission and was never a patient of this institution, which is a paediatric hospital; such documents that concern him reached the treating team only as papers supplied by the family and filed in the index patient’s chart. *CFTR* analysis was not performed in him because the test was not reimbursed, the family could not meet its cost, and a paediatric unit has no mechanism for funding investigation of an adult relative. Genetic testing was recommended to the family, but the recommendation could be carried no further than a referral. A CFTR-related disorder therefore remains possible but unconfirmed in the brother.

### 2.2. Diagnostic Work-Up at 12 Years of Age

In 2018, two sweat tests were performed with a Nanoduct system, which measures conductivity and reports the result as an NaCl-equivalent rather than as a chloride concentration. The values obtained were 125 and 80 mmol/L. Current guidance treats sweat conductivity as a screening measurement: a value of at least 50 mmol/L requires further evaluation, including *CFTR* analysis, whereas conductivity alone is not sufficient to establish the diagnosis [10]. Both measurements exceeded that threshold, but they are not equivalent in interpretive weight. A conductivity of 125 mmol/L lies within the range reported for classic cystic fibrosis with this device, whereas 80 mmol/L falls in the equivocal band between the exclusion and confirmation thresholds applied to Nanoduct readings [11,12]. Conductivity reflects the total ionic content of sweat rather than chloride alone and is sensitive to sweat rate, so a difference of this magnitude between two collections in the same individual does not by itself indicate analytical error; the collection conditions, the interval between the two measurements and the operator are not recorded in the documents available to us, so a technically compromised reading can be neither excluded nor demonstrated. Confirmatory chloride titration by the Gibson–Cooke or Macroduct method was not performed, and in retrospect, it should have been: the diagnosis in this patient rests on the higher value taken together with the genotype and the clinical phenotype, not on the pair.

Targeted *CFTR* analysis subsequently identified the 3849+10kbC>T variant together with a 5T poly-T allele. The 2018 report, as it appears in the records available to us, documents the presence of both findings and nothing further: it does not state the poly-T genotype of the second allele, the phase of the two findings, the analytical platform, or the extent of the region interrogated. Parental samples were not obtained and no allele-specific or long-range analysis was undertaken, so phase cannot be reconstructed retrospectively from what the document contains; the TG repeat adjacent to 5T was likewise not sized. We accordingly report the two findings as co-occurring rather than as demonstrably in *trans*. Compound heterozygosity is the configuration most consistent with the phenotype; it has not been demonstrated, and a *cis* configuration with a second, undetected CF-causing variant has not been excluded. The diagnosis of cystic fibrosis was made on the combination of a compatible clinical phenotype, two abnormal sweat conductivity values and the molecular findings, in accordance with the consensus criteria [9].

### 2.3. Interval Course, 2018–2023

Maintenance therapy comprised nebulised dornase alfa 2.5 mg once daily, pancreatic enzyme replacement (pancrelipase 25,000 IU three times daily, before main meals), ursodeoxycholic acid 250 mg twice daily, supplementation with the fat-soluble vitamins A, D, E and K, and a high-energy, high-fat diet targeting 110–200% of the recommended daily energy intake. Over the three years that followed, coinciding with the COVID-19 pandemic, documented exacerbations—defined here as episodes for which an antibiotic course was prescribed and recorded—numbered one to two per year. That figure should be read as a minimum: care during this period was dispersed between primary care, outpatient review and occasional admission, episodes managed outside this institution were not systematically captured, and the clinical impression at the time was of a higher burden than the documented count reflects. In the absence of spirometry, no independent measure of trajectory is available against which either figure could be checked. Most episodes were managed on an outpatient basis with oral and inhaled antibiotics selected from previous culture results—a pattern shaped less by clinical preference than by the restricted hospital access of the pandemic period.

Chronic *P. aeruginosa* infection with concurrent *S. aureus* was documented between August 2021 and March 2022. Inhaled colistimethate sodium was given as a dry-powder capsule, 1,662,500 IU twice daily, for six months, and achieved eradication: the isolate of March 2022 was the last positive culture, and surveillance cultures have remained negative since. *S. aureus* proved considerably more difficult. Oral ciprofloxacin, intravenous vancomycin and a 14-day course of intravenous teicoplanin 6 mg/kg/day each failed to clear the organism, persistence being confirmed on culture one month after the teicoplanin course. Intravenous agents during this interval were given at standard weight-based doses according to unit protocol; the individual doses are not recorded in the notes available for review and are therefore not reported here.

### 2.4. Index Admission (September 2023)

He was admitted to the Department of Paediatric Pulmonology, “Sf. Maria” Emergency Children’s Hospital, Iași, at 17 years of age, with a two-week history of productive cough followed by post-tussive vomiting. He was afebrile, with mildly affected general condition. Respiratory rate was 18/min and peripheral oxygen saturation 98% breathing room air. Body mass index was 21.2 kg/m^2^ (50th percentile), with sparse subcutaneous adipose tissue and no digital clubbing.

Spirometry was not performed. Local infection-control policy excludes patients with chronic *P. aeruginosa* or *S. aureus* airway infection from the shared pulmonary function laboratory, and no segregated facility is available; no earlier spirometric values are recorded either. Lung function is consequently uncharacterised at every point in this report, and we return to the implications of that in the Limitations.

Laboratory results at admission are shown in Table 2. The abnormalities were modest and internally consistent: C-reactive protein 11 mg/L and an erythrocyte sedimentation rate of 18 mm/h, with a haemoglobin marginally below the reference interval and a normal leucocyte count, a combination compatible with low-grade chronic airway inflammation rather than with acute bacteraemic infection; procalcitonin was undetectable. Creatinine lay at the lower limit, in keeping with lean body mass. Faecal elastase-1 exceeded 500 µg/g, confirming pancreatic sufficiency; enzyme replacement, started empirically at diagnosis, had nevertheless been continued for five years without formal reassessment. Hepatic enzymes and bilirubin were normal, as they had been throughout follow-up, ursodeoxycholic acid having likewise been continued without a documented indication.

**Figure 1:**
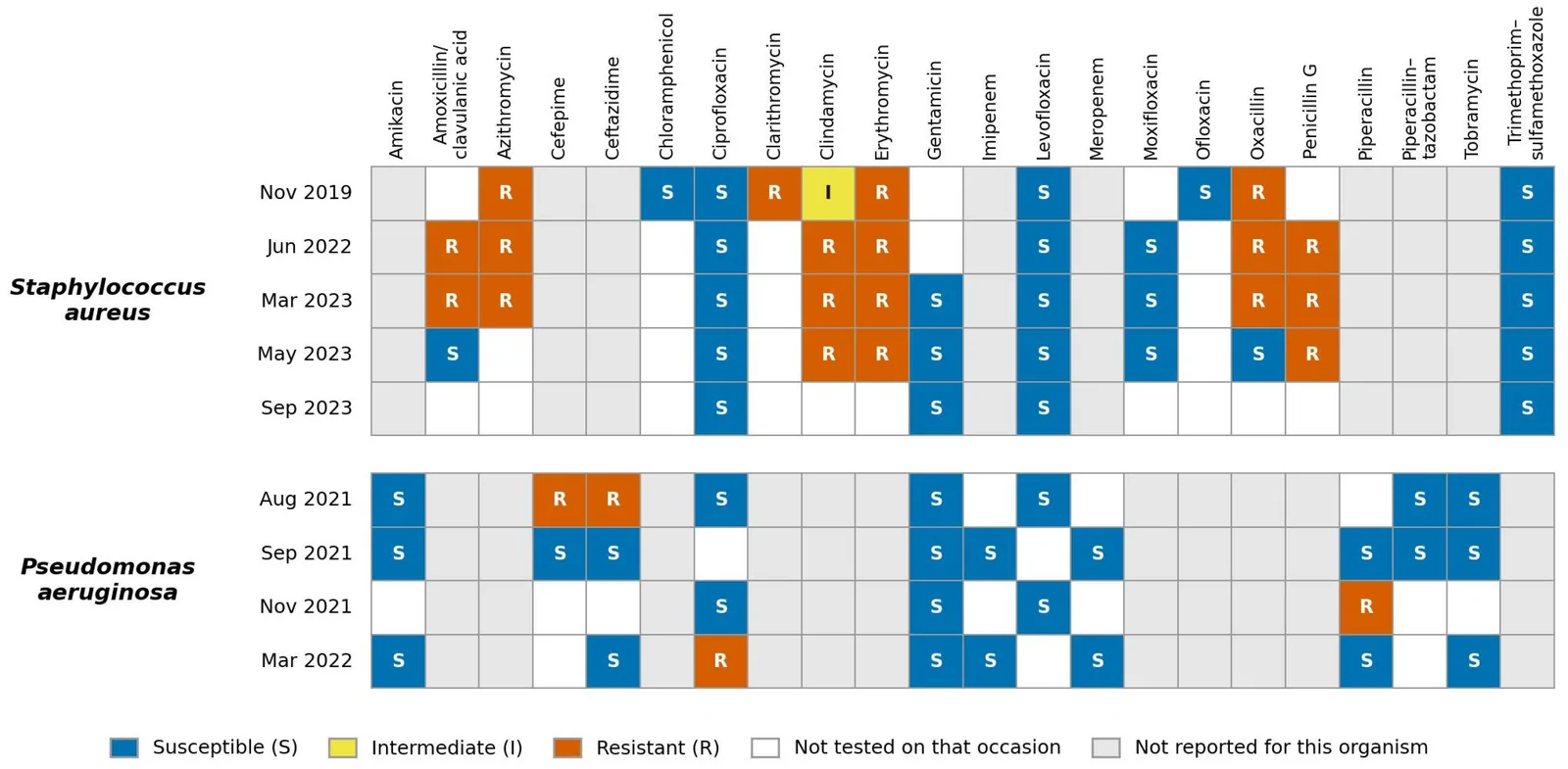
Antibiotic susceptibility of *Staphylococcus aureus* (upper panel) and *Pseudomonas aeruginosa* (lower panel) isolated from sputum cultures between November 2019 and September 2023, in chronological order within each organism. The September 2023 entry corresponds to the culture obtained during the admission described here; all preceding entries were obtained at outpatient visits or earlier hospitalisations. Susceptibility is shown as S, I or R within each cell, so that interpretation does not depend on colour; the palette has been chosen to remain distinguishable under deuteranopia. Cells shaded grey denote agents not reported for that organism, whether because the species is intrinsically resistant or because susceptibility is inferred from another marker, and were excluded from interpretation. White cells denote agents not tested on that occasion, and are the only category that indicates absence of data.

Chest computed tomography demonstrated bilaterally disseminated cylindrical and saccular bronchiectasis with mucoid impaction. Sputum culture grew *S. aureus*. Figure 1 shows the longitudinal susceptibility record for both organisms between November 2019 and September 2023, including the isolate obtained at this admission. The staphylococcal isolates were resistant to macrolides and to clindamycin on every occasion tested, and meticillin resistant on all but one of the occasions on which oxacillin was tested, the isolate of May 2023 being the single meticillin-susceptible exception; susceptibility to trimethoprim–sulfamethoxazole and to fluoroquinolones was retained throughout the period. The pseudomonal isolates showed variable susceptibility to cefepime and ceftazidime, consistent susceptibility to amikacin, gentamicin and tobramycin, and—a change worth noting—loss of ciprofloxacin susceptibility by the final isolate of March 2022, the organism having been susceptible in August and November 2021.

Empirical intravenous vancomycin and piperacillin–tazobactam, at standard weight-based doses, were started and de-escalated on day three, once susceptibility data became available, to oral levofloxacin 500 mg every 12 h for 14 days followed by trimethoprim–sulfamethoxazole 480 mg every 12 h for 14 days. Cough diminished from the second day of targeted treatment. No adverse events were recorded and hepatic function remained normal. Adherence to maintenance therapy was verified by structured interview and by pharmacy dispensing records. The full chronology of antimicrobial exposure is given in Table 3.

### 2.5. Genotype-Directed Therapy and Current Status

The 3849+10kbC>T variant is among the residual-function alleles for which in vitro responsiveness supports treatment with elexacaftor/tezacaftor/ivacaftor [13], an approach subsequently adopted in variant-specific standards of care [14]; extension of the label to such variants has been followed by measurable gains in lung function and by a marked reduction in exacerbation frequency in registry cohorts [13]. Reimbursed access for this patient was granted in January 2026, eight years after the diagnosis and seventeen years after his first respiratory symptoms. At the time of writing, therapy has been authorised but not yet followed up, so the clinical response cannot be reported here; the absence of any spirometric baseline means that, when it is, the response will have to be judged on exacerbation frequency and symptom burden alone.

Structured surveillance has been organised along established best-practice lines [15]: sputum culture at every visit, annual liver biochemistry with abdominal ultrasonography, annual oral glucose tolerance testing or glycated haemoglobin, periodic measurement of fat-soluble vitamin concentrations with review of enzyme replacement, and dual-energy X-ray absorptiometry at intervals appropriate to age and risk. Annual spirometry is part of that programme in principle; in practice, it awaits a segregated facility, and securing one has become the more pressing requirement now that a modulator response will need to be measured.

## 3. Discussion

### 3.1. What the Genotype Establishes, and What It Does Not

Two molecular findings were reported in this patient, and the distance between reporting them and interpreting them is the substance of this case. Neither the TG-repeat length in *cis* with 5T nor the phase of the two variants was determined. Both omissions matter, and they matter differently.

The TG haplotype decides whether 5T behaves as a low-penetrance background or as a functionally consequential allele [3]; without it, the genotype cannot be assigned a fixed position between CFTR-related disorder and non-classic cystic fibrosis under frameworks that require molecular completeness before a definite tier is issued [16]. Phase is the more consequential gap. Compound heterozygosity was assumed but never demonstrated: parental samples were not obtained, no family-based or long-range analysis was performed, and the coverage of the assay is undocumented. Should the two findings lie in *cis*, a second CF-causing variant would remain undetected and the molecular explanation of the phenotype would be incomplete. We therefore describe the genotype as co-occurring rather than compound heterozygous—a distinction that carries little weight for the patient’s immediate management, but a considerable one for the genetic counselling this family has been offered.

The phenotype is itself instructive. Pancreatic sufficiency, preserved nutritional status and the absence of chronic rhinosinusitis follow the pattern expected of a residual-function genotype. The sweat conductivity values sit less comfortably. A value of 125 mmol/L is high for a variant recognised precisely for producing normal or borderline results [2], whereas the second measurement, at 80 mmol/L, falls in the equivocal band [11,12]; the two therefore point in somewhat different directions, and the diagnosis rests on the higher of them taken together with the genotype. Whether the discordance reflects the unmeasured TG-T haplotype, an undetected third variant, a difference in collection conditions, or simply the width of individual variation around any genotype cannot be settled with the data in hand—which is, in itself, the point. Prediction of phenotype from genotype remains approximate at the level of the individual patient [1].

### 3.2. Sweat Conductivity and the Limits of a Negative Result

The distinction between conductivity and chloride operates in opposite directions in the two brothers. In the index patient, it was of no practical consequence: both measurements lay far above any published action threshold, and the genetic result settled the question. In the brother it is decisive. Two negative sweat tests were treated, in effect, as excluding cystic fibrosis. Conductivity below 50 mmol/L does carry reasonable negative predictive value in unselected populations [10], but that performance derives from cohorts in which residual-function genotypes form a small minority, and the very variant later identified in his sibling was first described in patients with normal sweat chloride [2]. A negative sweat test in a young adult with recurrent spontaneous pneumothorax and a genetically confirmed sibling is not a result that excludes CFTR involvement; it is a result that requires genotyping in order to be interpreted at all [14,17].

The presentation itself deserves separate emphasis. Recurrent spontaneous pneumothorax in a young adult who is also malnourished is not a self-explanatory event, and once a sibling has carried a confirmed *CFTR* genotype, it ceases to be an isolated finding altogether. The brother has still not been genotyped.

### 3.3. Which Components of the Delay Are Attributable to Screening

Symptoms began at three years; the diagnosis was made at twelve. Two explanations are usually offered for such an interval—absent newborn screening and restricted access to genetic testing—and they should not be given equal weight.

Screening was indeed unavailable in 2018 [7]. Whether it would have identified this child is a separate question, and the honest answer is that it might not have. Protocol sensitivity varies widely between programmes [8], and the pancreatic-sufficient, residual-function group is the one in which trypsinogen-based algorithms are least dependable [9]. We therefore do not claim that screening would have prevented this delay; we claim only that it would have shifted the probability.

The second explanation is better supported by the family’s own history. A sibling with recurrent spontaneous pneumothorax and malnutrition, in whom two sweat tests were negative, was never genotyped because the test was not reimbursed. Had he been genotyped, cystic fibrosis or a CFTR-related disorder would at least have entered a differential in which no such possibility had been raised. Whether the index diagnosis would then have been reached sooner cannot be established, and we advance it as a plausibility rather than as an inference. The documented fact is narrower and sufficient: the test was not performed, the reason recorded was cost, and it has still not been performed. The resource whose absence is documented here is not a population screening programme but a molecular test in a symptomatic relative—cheaper, more targeted and never performed. Surveys of European screening programmes and of global CF care describe the same disproportion between diagnostic capability and diagnostic access, which suggests a structural rather than a local failure [8,18].

The discontinuity is itself worth naming. A paediatric service can identify the genetic implication of an index diagnosis for an adult sibling, and can recommend that it be pursued, but it has no route by which to pursue it: the brother was never a patient of this institution, and such documents that concerned him reached the treating team only through the family. Whether the test is then performed depends on the family’s means. In this family it was not performed in 2018, and it has not been performed since.

### 3.4. From Genotype to Treatment Eligibility

For most of the interval described here, complete genotyping would have altered counselling without altering treatment. That is no longer true. Approval of elexacaftor/tezacaftor/ivacaftor for a defined set of rare variants on the basis of in vitro responsiveness, rather than variant-specific trials, converted the *CFTR* genotype from a diagnostic label into an access criterion [13,14,19]. Registry data following the expanded label indicate meaningful improvement in lung function and a marked reduction in exacerbations among people carrying no F508del allele [13], and the sustained durability of these clinical benefits has been clearly documented in long-term extension studies [20]. Modulator therapy also reduces airway bacterial burden, although eradication of established chronic infection is not the expected outcome and should not be presented to patients as one [21].

The practical implication for this patient is direct: without the 2018 genotype he would not have been eligible in 2026. The implication for his brother is equally direct and remains unrealised. Where access to modulators depends on a documented variant, genotyping ceases to be a confirmatory step and becomes the gateway to treatment—an argument for reimbursing it that does not rest on diagnostic yield alone.

### 3.5. Antimicrobial Management in Retrospect

Most exacerbations in this patient were treated orally, which registry analyses identify as the predominant pattern in children and adolescents managed predominantly in the outpatient setting, roughly one fifth of whom subsequently require intravenous therapy or admission [22,23]. Early eradication of *P. aeruginosa* rests on the firmest evidence base of any antimicrobial intervention in cystic fibrosis, and the six-month inhaled colistimethate course achieved a durable result, cultures remaining negative for more than three years [24,25]. *S. aureus* was the harder problem, as it usually is: eradication protocols for meticillin-resistant isolates are of uncertain benefit, and successive courses of vancomycin, teicoplanin and fluoroquinolones failed here much as they fail elsewhere [24].

Two aspects invite criticism, and we prefer to raise them ourselves. Sequential oral fluoroquinolone therapy directed at *S. aureus* carries a recognised risk of selecting resistance, and the combination regimens with better support—trimethoprim–sulfamethoxazole with rifampicin, for instance—were not employed [24]. The susceptibility record is at least suggestive: *P. aeruginosa*, susceptible to ciprofloxacin in August and November 2021, was resistant by the isolate of March 2022, over an interval during which oral fluoroquinolone was among the agents in repeated use. We cannot demonstrate that the two are causally linked—the isolates were not typed, and loss of susceptibility may equally reflect strain replacement—but the temporal association is close enough to be worth stating. Second, therapy was pursued along susceptibility results throughout, although in vitro susceptibility predicts clinical response inconsistently in chronic, biofilm-associated airway infection, where the organism’s behaviour in the sputum bears little relation to its behaviour on an agar plate [24,26,27]. In a patient with established bronchiectasis, persistent *S. aureus* and no available spirometry, the endpoint against which each successive course should have been judged was never defined objectively. That is a limitation of the care delivered, not merely of its documentation.

Two maintenance drugs were continued without periodic re-evaluation. Ursodeoxycholic acid was given for years in a patient whose hepatic biochemistry and abdominal ultrasonography remained normal throughout, a practice against which current consensus recommendations advise explicitly [28]; pancreatic enzyme replacement, started empirically at diagnosis, was maintained for five years in a patient whose faecal elastase-1 exceeded 500 µg/g. Neither was harmful. Both added to a treatment burden that the introduction of modulator therapy makes it timely to reconsider.

### 3.6. Limitations

Several limitations follow from the same constraint. This report reconstructs eight years of care from documents held by a paediatric service, some of which originated elsewhere and reached the chart through the family. The original molecular report is incomplete as to platform, coverage and poly-T genotype, so that the TG-repeat length, the phase of the two variants and the parental genotypes are all unknown and an unexamined third variant cannot be excluded; the brother’s sweat test values and method are unavailable; the conditions under which the two conductivity measurements were obtained are unrecorded; and exacerbations managed outside this institution were not systematically captured. Lung function is uncharacterised at every point, because infection-control policy excluded the patient from the shared pulmonary function laboratory and no segregated facility existed; progression of the bronchiectasis therefore cannot be quantified, and the response to modulator therapy will have no baseline against which to be measured. Elements of routine annual review—oral glucose tolerance testing, fat-soluble vitamin concentrations, mycobacterial culture, *Aspergillus* serology and bone densitometry—are absent from the record [15]. Finally, modulator therapy has been approved but not yet followed up, so the outcome that would most interest the reader is the one we cannot supply. We report these gaps rather than estimate around them, and we note that most of them are of a kind: the record of a patient whose care crossed services, institutions and reimbursement boundaries is fragmentary in precisely the places where those boundaries lie.

## 4. Conclusions

The interest of this case does not lie in the rarity of the genotype, which is catalogued and well described. It lies in the unequal weight that a single molecular test carried at two points in the same life. At diagnosis it was one element among three, alongside a compatible clinical phenotype and two abnormal sweat conductivity values, and the diagnosis is properly attributed to their combination. Eight years later it was not one element among several but the sole criterion: in the absence of a documented variant, elexacaftor/tezacaftor/ivacaftor could not have been prescribed at all. The same test, never performed in his symptomatic brother, might have brought cystic fibrosis or a CFTR-related disorder into a differential from which it was absent for nine years; whether the index diagnosis would have followed sooner is a counterfactual we cannot test, and we do not assert it. What is not counterfactual is that the brother remains ungenotyped, and that completion of the molecular picture—TG-repeat determination and parental studies in the index patient, *CFTR* analysis in his brother—is the most useful intervention still available to this family.

## Figures and Tables

**Table 1:** Chronology of clinical events.

Date (Age)	Event
2009 (3 years)	Onset of recurrent upper and lower respiratory tract infections
Before 2018	Elder brother investigated for recurrent spontaneous pneumothorax and malnutrition; two negative sweat tests; *CFTR* analysis not performed (not reimbursed)
2018 (12 years)	Two sweat conductivity measurements, 125 and 80 mmol/L NaCl-equivalent; targeted *CFTR* analysis identifying 3849+10kbC>T and 5T; diagnosis of cystic fibrosis; maintenance therapy started
2019–2022	One to two documented episodes per year (see text); predominantly treated with oral and inhaled antibiotics in the outpatient setting
August 2021–March 2022	Chronic *P. aeruginosa* infection with concurrent *S. aureus*; six months of inhaled colistimethate; last positive pseudomonal culture March 2022, surveillance cultures negative thereafter; *S. aureus* persistent despite ciprofloxacin, vancomycin and teicoplanin
July 2022	Newborn screening for cystic fibrosis introduced as national policy in Romania
September 2023 (17 years)	Index admission; bilateral cylindrical and saccular bronchiectasis on computed tomography; *S. aureus* in sputum; spirometry not performed (infection-control policy)
January 2026 (20 years)	Elexacaftor/tezacaftor/ivacaftor approved on the basis of the genotype; no follow-up data yet

**Table 2:** Laboratory findings at the index admission.

Parameter	Result	Reference Interval
Haemoglobin	**12.8 g/dL**	13.0–16.0
Haematocrit	38.4%	36–46
Leucocytes	9.8 × 10^9^/L (neutrophils 65%, lymphocytes 28%)	4.5–13.0 × 10^9^/L
Platelets	310 × 10^9^/L	150–400 × 10^9^/L
C-reactive protein	**11 mg/L**	<5
Erythrocyte sedimentation rate	**18 mm/h**	<15
Procalcitonin	<0.05 ng/mL	<0.5
AST/ALT/GGT	24/19/14 U/L	<40/<41/<55
Total bilirubin	0.5 mg/dL	0.2–1.2
Urea/creatinine	24 mg/dL/**0.42 mg/dL**	15–40/0.50–1.00
Amylase/lipase	36/21 U/L	28–100/13–60
Faecal elastase-1	>500 µg/g	>200 (pancreatic sufficiency)
Stool studies	Negative	No *Clostridioides difficile* toxin, parasites or fungi

Reference intervals are those reported by the performing laboratory for the patient’s age and sex. Values falling outside the reference interval are shown in bold.

**Table 3:** Chronology of antimicrobial therapy.

Period	Indication	Regimen
August 2021–March 2022	Chronic *P. aeruginosa* infection with concurrent *S. aureus*	Intravenous meropenem or ceftazidime with amikacin, and intravenous vancomycin or linezolid, at standard weight-based doses; inhaled colistimethate sodium 1,662,500 IU twice daily for 6 months
After March 2022	Persistent *S. aureus* after ciprofloxacin and vancomycin	Intravenous teicoplanin 6 mg/kg/day for 14 days; culture remained positive one month later
Index admission, days 1–3	Empirical coverage pending susceptibility results	Intravenous vancomycin with piperacillin–tazobactam at standard weight-based doses
Index admission, from day 3	De-escalation guided by susceptibility testing	Levofloxacin 500 mg every 12 h for 14 days, then trimethoprim–sulfamethoxazole 480 mg every 12 h for 14 days

## Data Availability

The data supporting the findings of this report are contained within the article; further details are available from the corresponding author on reasonable request, within the limits imposed by patient confidentiality.

## Abbreviations

BMI, body mass index; CF, cystic fibrosis; CFTR, cystic fibrosis transmembrane conductance regulator; CRP, C-reactive protein; CT, computed tomography; ETI, elexacaftor/tezacaftor/ivacaftor; IRT, immunoreactive trypsinogen; MRSA, meticillin-resistant *Staphylococcus aureus*; NBS, newborn screening.
